# Revised Household-Based Microplanning in Polio Supplemental Immunization Activities in Kano State, Nigeria. 2013–2014

**DOI:** 10.1093/infdis/jiv589

**Published:** 2016-04-02

**Authors:** Emmanuel Gali, Pascal Mkanda, Richard Banda, Charles Korir, Samuel Bawa, Charity Warigon, Suleiman Abdullahi, Bashir Abba, Ayodeji Isiaka, Yared G. Yahualashet, Kebba Touray, Ana Chevez, Sisay G. Tegegne, Peter Nsubuga, Andrew Etsano, Faisal Shuaib, Rui G. Vaz

**Affiliations:** 1 World Health Organization, Country Representative Office, Abuja, Nigeria; 2 World Health Organization, Regional Office for Africa, Brazzaville, Congo; 3 Global Public Health Solutions, Atlanta, Georgia; 4 National Primary Health Care Development Agency, Abuja, Nigeria

**Keywords:** household-based microplanning, chronically missed children, workload rationalization, polio supplemental immunization activities

## Abstract

***Background.*** Remarkable progress had been made since the launch of the Global Polio Eradication Initiative in 1988. However endemic wild poliovirus transmission in Nigeria, Pakistan, and Afghanistan remains an issue of international concern. Poor microplanning has been identified as a major contributor to the high numbers of chronically missed children.

***Methods.*** We assessed the contribution of the revised household-based microplanning process implemented in Kano State from September 2013 to April 2014 to the outcomes of subsequent polio supplemental immunization activities using used preselected planning and outcome indicators.

***Results.*** There was a 38% increase in the number of settlements enumerated, a 30% reduction in the number of target households, and a 54% reduction in target children. The reported number of children vaccinated and the doses of oral polio vaccine used during subsequent polio supplemental immunization activities showed a decline. Postvaccination lot quality assurance sampling and chronically missed settlement reports also showed a progressive reduction in the number of children and settlements missed.

***Conclusions.*** We observed improvement in Kano State's performance based on the selected postcampaign performance evaluation indicators and reliability of baseline demographic estimates after the revised household-based microplanning exercise.

The 41st World Health Assembly in 1988 resolved to launch the Global Polio Eradication Initiative aimed at achieving a polio-free world by the year 2000 [1, 2]. Remarkable progress has been made, with a reduction from 350 000 cases in 125 countries in 1988 to 223 cases in 5 countries by the end of 2012 [3, 4]. In May 2012, the 65th World Health Assembly declared the completion of polio eradication a programmatic emergency for global public health [5–7]. At that time endemic wild poliovirus (WPV) transmission in 3 countries—Nigeria, Pakistan, and Afghanistan—constituted a risk factor for WPV reintroduction to polio-free countries and an obstacle to global polio eradication [8, 9]. Nigeria reported 122 cases of WPV in 2012, of which 28 (23%) were from Kano State in northern Nigeria [3, 10]. Kano State has traditionally been regarded as the epicenter of polio transmission in Nigeria and was the origin of the boycott of polio vaccination campaigns in 2003 [11]. Poliovirus of Nigerian and Kano State origin has been implicated as the source of reinfection to 25 polio-free countries since 2003 [6, 12].

The 2012 Nigeria National Polio Eradication Emergency Plan (NPEEP) included in its priorities the need to significantly reduce the numbers of chronically missed children by strengthening microplanning [13]. The Sixth Independent Monitoring Board of the Global Polio Eradication Initiative report in June 2012 identified poor microplanning as a major impediment to achieving the implementation of quality polio supplemental immunization activities (SIAs) [8]. Microplanning for polio SIAs is a process of estimation of the target populations and resource requirements for conduct of good-quality implementation in a specified location.

The guidelines for microplanning for polio SIAs in Nigeria were revised to focus on the target number of households rather than the target number of children per vaccination team in August 2012. The revised guidelines were piloted in 87 very high-risk (VHR) local government areas (LGAs) in the 11 VHR states that were identified based on their risk categorization using the combined US Centers for Disease Control and Prevention–Global Goods Criteria [14]. The 24th Expert Review Committee on Polio Eradication in Nigeria in September 2012 recommended the expansion of implementation to all the LGAs in the 11 VHR states and the intensification of oversight of the 2012 NPEEP [13]. This resulted in the establishment of the National Emergency Operations Center (EOC) by the presidential task force and subsequent establishment of EOCs in 5 states.

In August 2013, the Kano State EOC identified inflation of target population estimates with its consequent increase in requests for vaccines and high number of missed settlements as areas of serious concern in the available microplans; and with orientation, guidance and support from the country team organized a revised household-based microplanning exercise including microcensus (a process of estimating the numbers of households and eligible children in a given area by physical count, enumeration, and revalidation) was initiated. This process was aimed at collecting reliable demographic information on the target numbers of households and children in the communities. This article describes the process of implementing revised household-based microplanning intervention in Kano State between September 2013 and April 2014 and highlights the contributions of revised microplanning to improved outcomes of polio SIAs.

## METHODS

### Intervention Area and Population

The revised household-based microplanning intervention was implemented from September 2013 to April 2014 in 44 LGAs in Kano State with a total population of 12 568 289 based on the 2006 population census projections.

### Microplanning Strategy

Revised household-based microplanning was conducted in 2 phases for effective supervision. Phase 1 involved 19 LGAs in the southern part of the state where there was an ongoing polio outbreak at the time (September 2013), and phase 2 involved the remaining 25 LGAs from the central and northern parts of the state.

The microplanning process had 6 stages: (1) preparatory stage, (2) fieldwork, (3) revalidation, (4) workload rationalization, (5) feedback to key stakeholders, and (6) continuous microplan updating. The preparatory stage consisted of planning activities, meetings, and discussions on the revised microplanning concepts at the Kano State EOC and training of microplan supervisors and enumerators at the state, LGA, and ward levels. Desk review of the old microplans, daily implementation plans, and harmonization of master list of settlements from various sources were also conducted. During this stage budgetary considerations were finalized, and all data tools and materials needed for conducting the project were provided. During the fieldwork stage, physical walk-throughs were conducted in the catchment areas, including microcensus with enumeration of the total number of households and total numbers of eligible children <5 and <1 year of age in all the households in the catchment settlements. There was also concurrent drawing of daily route maps, catchment area demarcation, and daily implementation plans.

The revalidation process was designed to build confidence in the data generated from the revised microplans and confirmed that physical walk-throughs actually took place and enumeration data generated from this process was used to develop the daily implementation plans. During this stage, external teams of senior supervisors were deployed to revalidate the data from the microplan enumeration and the developed catchment area maps using geographical information system maps. The process was conducted in 15 of the 44 LGAs, selected based on their VHR categorization using the combined Centers for Disease Control and Prevention–Global Goods Criteria, persistent poor performance in the previous 12 months, and huge variance in target population and numbers of enumerated households before and after the revised microplanning exercise. Ten percent of the teams in the selected LGAs were randomly selected for revalidation of 1 randomly selected day of the team's work area, and based on the variance in findings the microplans were either accepted or rejected. LGAs with <20% variance were accepted; those with variance of >20% to <30% were revalidated for an additional 10 more teams and were rejected if the result was the same or worse. LGAs with >40% variance when 50% of the wards had been revalidated were also rejected. All rejected LGAs had to repeat the entire process.

In the workload rationalization stage, accepted and revalidated microplans were assessed to ensure that the daily workload of the teams was manageable and realistic depending on the settlement profiles based on the microplanning guidelines. To provide feedback to key stakeholders at the end of the revised household-based microplanning intervention and validation, the outcomes were shared with the vaccination team supervisors, ward focal persons, LGA teams and EOCs at state and national levels.

Finally, for continuous updating, the developed microplans were updated by an inbuilt microplan updating process that involved concurrent household enumerations during implementation of polio SIAs and tally sheet analysis after each round of SIAs, and the new information obtained was used to update the microplans. Reports of settlement validations on reported “missed” settlements using tablets to verify machine-generated and named hamlet areas or settlements are also constantly used to update the microplans.

### Data Collection and Analysis

The data considered during the conduct of the intervention were the direct outputs of the microplanning process, including the numbers of settlements, households, and eligible children; the numbers of teams engaged and average team workload; outcome indicators from postcampaign performance evaluation data; lot quality assurance sampling (LQAS) performance trends; and geographical information system missed settlement reports. The data were obtained from the Kano State EOC, with postcampaign performance evaluation data from the World Health Organization Nigeria data unit and the website of the Nigeria Vaccination Tracking System. To determine the contribution of the revised microplanning process to the outcome of polio SIAs implementation, the values obtained for the selected data and indicators were compared for polio SIAs implemented before and after the implementation of the revised microplanning project.

## RESULTS

There was an increase in the number of settlements in the master list from 20 388 to 28 084 (a 38% increase), a reduction in the number of households from 2 491 405 to 1 736 898 (a 30% reduction), and a reduction in target children from 6 087 511 to 2 807 551 (a 54% reduction) after the enumeration. The average number of households and target children allocated to the house to house teams after workload rationalization was reduced from 94 to 65 households (a 31% reduction) and from 231 to 106 target children (a 54% reduction) per team per day (Table 1).

**Table 1.** Key Process Output Before and After the Revised Household-Based Microplanning Process in Kano State, September 2013 and April 2014

Key Outputs	Before Microplanning (September 2013)	After Microplanning (April 2014)	Change in Output, %
Identified settlements, No.	20 338	28 074	38
Enumerated households, No.	2 491 405	1 736 898	−30
Target population (age <5 y), No.	6 087 511	2 807 551	−54
House-to-house teams, No.	6596	6630	1
Households/team/d, No.	94	65	−31
Target population/team/d, No.	231	106	−54

**Figure 1.**
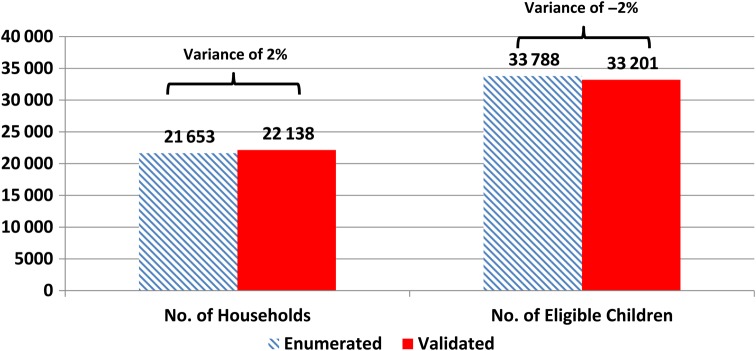
Comparison between number of households enumerated during the microplanning exercise and during the revalidation exercise in the 15 local government areas selected after the microplanning exercise in Kano State.

**Figure 2.**
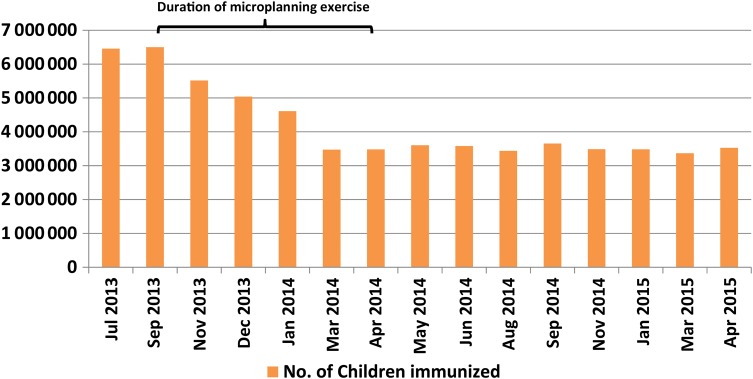
Trend in absolute number of eligible children immunized during the polio supplemental immunization activities conducted in Kano State from July 2013 to April 2015.

Data from the validation process showed an increase in the numbers of households after revalidation from 21 653 to 22 138 (2% variance) and a reduction in the numbers of eligible children in the households from 33 788 to 33 201 (−2% variance) (Figure 1). The number of children vaccinated during the polio SIAs started declining in September 2013 when the revised microplanning intervention began, and this trend continued until March 2014. The trend stabilized in April 2014, with slight variations from one round of polio SIA to the next. A total of 6 499 164 children were vaccinated in September 2013, and this number decreased to 3 480 593 by April 2014 (46% reduction). During the April 2015 polio SIA, 3 525 562 children were vaccinated (Figure 2).

**Figure 3.**
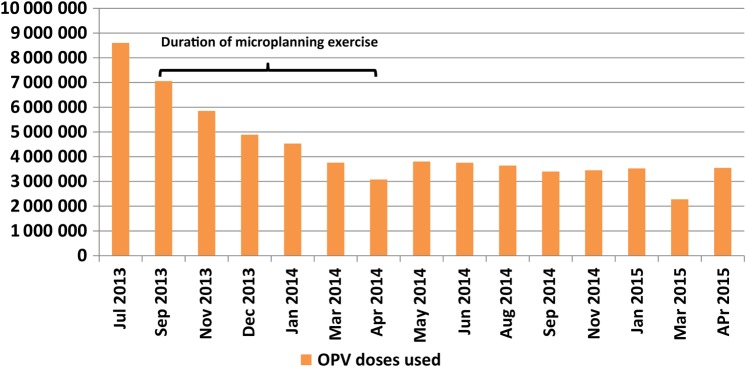
Doses of oral polio vaccine (OPV) used to vaccinate eligible children during the polio supplemental immunization activities conducted in Kano State from July 2013 to April 2015.

The doses of oral polio vaccine (OPV) used to vaccinate children during the polio SIAs started declining in September 2013 when the revised microplanning started; this trend continued until April 2014, and then the numbers became fairly stable from May 2014, with slight variations. The doses used decreased from 7 054 880 doses in September 2013 to 3 076 580 by April 2014 (a 56% reduction). During the April 2015 polio SIA, 3 547 760 doses were used. Vaccine utilization data from September 2014 to April 2014 revealed that a total of 13 165 320 doses of OPV were saved by April 2014, compared with the vaccine utilization in September 2013. As of April 2015, a total of 42 196 600 doses of OPV had been saved (Figure 3).

The trends in LQAS performance of the state improved; before the revised microplanning project began (in September 2013), 24% of the LGAs surveyed were accepted at ≥90%, whereas by April 2014, when the project was concluded, the percentage of LGAs accepted at ≥90% had increased to 66%. From April 2014 onward, the percentage of LGAs accepted at >90% has consistently improved (range, 66%–92%). In April 2015, this figure was 84% (Table 2).

**Table 2.** Trends in LQAS Performance of the LGAs in Kano State From April 2013 to April 2015

LQAS Performance, %^a^	LGAs Surveyed, No. (%)																		
	2013							2014									2015		
	April	May	June	July	September	November	December	January	March	April	May	June	August	September	November	December	January	March	April
Total	24	24	24	22	17	36	36	37	39	38	39	39	39	39	44		44	44	44
≥90	6 (25)	6 (25)	4 (17)	4 (18)	4 (24)	10 (28)	15 (42)	26 (70)	30 (77)	25 (66)	31 (79)	33 (85)	33 (85)	36 (92)	29 (66)	33 (75)	37 (84)	32 (73)	37 (84)
80–89.9	8 (33)	10 (42)	6 (25)	6 (27)	8 (47)	13 (36)	11 (31)	11 (30)	6 (15)	12 (32)	74 (18)	6 (15)	6 (15)	3 (8)	14 (32)	9 (20)	6 (14)	8 (18)	7 (16)
60–79.9	7 (29)	7 (29)	11 (46)	10 (45)	5 (29)	12 (33)	9 (25)	0 (0)	3 (8)	1 (3)	1 (3)	0 (0)	0 (0)	0 (0)	1 (2)	2 (5)	1 (2)	2 (5)	0 (0)
<60	3 (13)	1 (4)	3 (13)	2 (9)	0 (0)	1 (3)	1 (3)	0 (0)	0 (0)	0 (0)	0 (0)	0 (0)	0 (0)	0 (0)	0 (0)	0 (0)	0 (0)	2 (5)	0 (0)

Abbreviations: LGAs, local government areas; LQAS, lot quality assurance sampling.

^a^ LQAS performance was defined as followed: 0–3 missed children, ≥90%; 4–8 missed children, 80%–89.9%; 9–19 missed children, 60%–79.9%; and >19 missed children (<60%).

**Figure 4.**
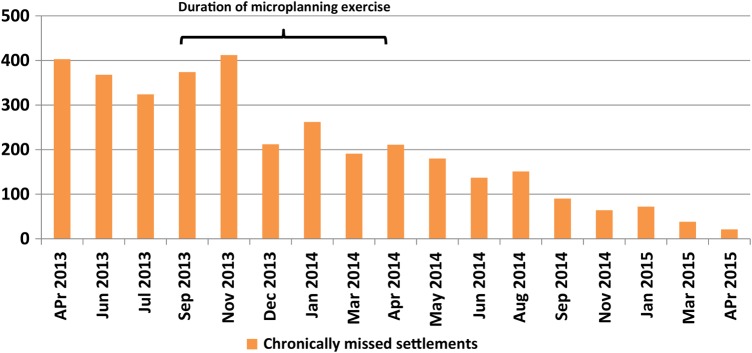
Absolute numbers of chronically missed settlements in Kano State from April 2013 to April 2015.

The number of chronically missed settlements (settlements that have been missed consistently for ≥3 consecutive rounds of polio SIAs) was reduced progressively from November 2013 after the revised microplanning project began. These numbers were high before the revised microplanning process began. In September 2013, there were 374 chronically missed settlements, and these numbers were reduced to 211 by April 2014 and 21 by April 2015, showing consistent reduction (Figure 4). An improvement was observed in Kano State's performance after initiation of the revised microplanning process, based on the selected postcampaign performance evaluation indicators and reliability of baseline demographic estimates such as target populations, households, and team workload.

## DISCUSSION

Our revised microplanning intervention for polio SIAs in Kano State revealed that that the numbers of target households and eligible children used in the previous microplans were indeed inflated. We also identified missed settlements during the revised microplanning process. This supported the observations of the Sixth Independent Monitoring Board of the Global Polio Eradication Initiative report in June 2012 that the existing microplans were faulty [8]. Our intervention confirmed the concerns of the Kano State EOC on the reliability of the data estimates on target populations, children immunized, and missed settlements from administrative reporting and the assertion in the 2012 NPEEP on the need to strengthen the available microplans to reduce the number of missed children [13].

We also found that the reduction in the target population of eligible children also translated into a reduction in overreporting on the numbers of children immunized during subsequent polio SIAs. There was a consistent reduction in the number of children immunized from the onset of the revised microplanning process in September 2013 to April 2014 when the revised microplans were concluded. This reduction had an impact on the quantities of vaccine and other materials used and enabled cost savings.

Implementation of subsequent polio SIAs using the daily implementation plans derived from the revised microplanning exercise showed improved outcomes based on postcampaign performance evaluation. Analysis of the results of LQAS conducted in the SIAs implemented after the completion of the revised microplanning exercise demonstrated an increase in the proportion of LGAs surveyed accepted at ≥90%, indicating improved outcome. Postcampaign Vaccination Tracking System reports on chronically missed settlements showed a consistent decrease in the numbers of missed settlements since the completion of the revised microplanning exercise, giving further credence to the benefits derived from the revised microplanning exercise for improved outcomes of polio SIAs.

The revalidation process of the revised microplanning exercise gave credibility to the data generated from the process. The output of the revised microplanning exercise in Kano State was validated as accepted for use in the workload rationalization and final development of the daily implementation plans.

The limitations of our approach include the cost of implementing the household-based microplanning project, which requires funds for payment of personnel, transportation, and production of tools. The revalidation process also had its limitations in the proportion of microplans revalidated, which is also related to cost of engaging adequate numbers of external validators. However, we believe that the cost savings from additional vaccines and the public health impact of identifying and vaccinating chronically missed children justify the extra cost of implementing this approach. Another additional limitation is that several other interventions were concurrently implemented during the microplanning period, and some of the improved outcome demonstrated by this project may be equally attributable to the other interventions.

Despite these potential limitations, the revised household-based microplanning is essential if any improvement in the quality of polio SIAs is to be realized and sustained. A good microplan ensures improved team performance, workload rationalization with its resultant reduction in missed settlements and children, and reduced overreporting of children immunized. The microcensus data on the number of children <1 year old has been applied to routine immunization microplanning and a project on vaccinating hard-to-reach children in Kano state.

We uphold the recommendation of the 24th Expert Review Committee on Polio Eradication in Nigeria on expanding the revised household-based microplanning process to all VHR states. The importance of conducting household-based microplanning to reduce the numbers of missed settlements, households, and eligible children during polio SIAs, toward the ultimate goal of reaching the very last child with polio vaccines, cannot be overemphasized. Critical to the successful implementation of this activity, however, are the adaptation of the existing guidelines to address local peculiarities; adequate planning for engagement, training, and provision of logistics for the enumerators; and adequate supervision and revalidation by senior supervisors.
